# Characterization of NR1J1 Paralog Responses of Marine Mussels: Insights from Toxins and Natural Activators

**DOI:** 10.3390/ijms25126287

**Published:** 2024-06-07

**Authors:** Antonio Casas-Rodríguez, Concepción Medrano-Padial, Angeles Jos, Ana M. Cameán, Alexandre Campos, Elza Fonseca

**Affiliations:** 1 Area of Toxicology, Faculty of Pharmacy, Universidad de Sevilla, Profesor García González n◦2, 41012 Seville, Spain; acasasr@us.es (A.C.-R.); angelesjos@us.es (A.J.); camean@us.es (A.M.C.); 2 Laboratorio de Fitoquímica y Alimentos Saludables (LabFAS), Centro de Edafología y Biología Aplicada del Segura, Consejo Superior de Investigaciones Científicas (CEBAS-CSIC), Campus Universitario 25, Espinardo, 30100 Murcia, Spain; 3 Centro Interdisciplinar de Investigação Marinha e Ambiental (CIIMAR/CIMAR), University of Porto, Terminal de Cruzeiros do Porto de Leixões, Av. General Norton de Matos, s/n, 4450-208 Matosinhos, Portugal; fonseca.ess@gmail.com

**Keywords:** pregnane X receptor, NR1J1 paralogs, bivalves, cyanotoxins, toxins, microalgal extracts, marine mussels

## Abstract

The pregnane X receptor (PXR) is a nuclear hormone receptor that plays a pivotal role in regulating gene expression in response to various ligands, particularly xenobiotics. In this context, the aim of this study was to shed light on the ligand affinity and functions of four NR1J1 paralogs identified in the marine mussel *Mytilus galloprovincialis*, employing a dual-luciferase reporter assay. To achieve this, the activation patterns of these paralogs in response to various toxins, including freshwater cyanotoxins (Anatoxin-a, Cylindrospermopsin, and Microcystin-LR, -RR, and -YR) and marine algal toxins (Nodularin, Saxitoxin, and Tetrodotoxin), alongside natural compounds (Saint John’s Wort, Ursolic Acid, and 8-Methoxypsoralene) and microalgal extracts (*Tetraselmis*, *Isochrysis,* LEGE 95046, and LEGE 91351 extracts), were studied. The investigation revealed nuanced differences in paralog response patterns, highlighting the remarkable sensitivity of MgaNR1J1γ and MgaNR1J1δ paralogs to several toxins. In conclusion, this study sheds light on the intricate mechanisms of xenobiotic metabolism and detoxification, particularly focusing on the role of marine mussel NR1J1 in responding to a diverse array of compounds. Furthermore, comparative analysis with human PXR revealed potential species-specific adaptations in detoxification mechanisms, suggesting evolutionary implications. These findings deepen our understanding of PXR-mediated metabolism mechanisms, offering insights into environmental monitoring and evolutionary biology research.

## 1. Introduction

Nuclear hormone receptors (NRs) are transcription factors associated with co-factors that regulate gene expression, usually upon binding to a ligand [1]. They are proteins exclusive to metazoans and expressed in different tissues [2]. Seven subfamilies have been identified in mammals, each performing varied molecular and physiological functions, encompassing gene regulation and intracellular metabolic and physiological homeostasis, as well as orchestrating cellular differentiation and developmental processes [3,4,5]. The current hypothesis suggests that NRs emerged early in animal evolution from ancestral receptors that first appeared in invertebrates evolving through a series of gene amplifications and subsequent mutations and diversifications [6,7]. This theory is consistent with the absence of several NR orthologous genes in invertebrates, such as some vertebrate endocrine hormone targets (e.g., androgen receptor AR, glucocorticoid receptor GR) from subfamily 3 group C [8]. NRs are part of the endocrine system regulating and coordinating multiple processes involved in lipid and cholesterol metabolism and bile salt synthesis [8], in addition to playing a critical role in embryonic and post-embryonic development [9,10]. Hence, the impairment of NR signaling has been related to several metabolic and inflammatory diseases [3,11].

Most NRs share a conserved modular structure that includes, from the N-terminus to the C-terminus, the modulatory A/B domain, the DNA-binding domain (DBD, C domain), the ‘hinge’ D domain, the ligand-binding domain (LBD, E domain), and a variable C-terminal F domain that can be absent in some NRs [6,12]. The DBD mediates NR binding to DNA sequence-specific response elements. DBDs are highly conserved among species, comprising about 80 amino acids that fold to form two zinc fingers, each composed of four cysteine residues that chelate a zinc atom. In turn, LBDs mediate ligand recognition and binding, consisting of approximately 250 amino acids that fold into a hydrophobic pocket where ligands bind [12].

The pregnane X receptor (PXR) belongs to subfamily 1 group I (NR1I2) [13] and is considered a key element in the defense against toxic substances, including foreign chemicals (xenobiotics) [14]. The name ‘pregnane’ X receptor came from its activation by pregnane (21-carbon or C21) steroids such as progesterone or 5β-pregnan-3,20-dione [14]. PXR exerts a significant influence on metabolism modulation, cell cycle arrest, inflammation, and angiogenesis [15]. Moreover, it regulates and coordinates xenobiotic metabolism, which includes oxidation and conjugation reactions and the transport of metabolites (xenobiotics) [12]. Given these functions, PXR is a primary xenobiotic sensor with a critical role in protecting against chemical challenges. Compared to other NRs, PXR modulates a broad spectrum of biological processes, and the differences among orthologous sequences are consistent with the extraordinary differences in PXR ligand specificities across vertebrate species, supporting the theory that PXR evolved to adapt to cross-species differences in exogenous and endogenous toxic compounds [14,16]. Among the endogenous and exogenous chemicals that activate vertebrate PXR are steroids, bile acids, environmental pollutants, and prescription drugs [12]. Mammalian and vertebrate PXRs regulate genes from phases I (*cyp*3A family members, *cyp*2B6, *cyp*2b9, *cyp*2C8, *cyp*2C9, and *cyp*2C19), II (glutathione-S-transferase, sulfotransferase, UDP-glucuronosyltransferase, and carboxylesterase family genes), and III (hepatic transporters *oatp*2, *mrp*2, and *mdr*1) of xenobiotic detoxification [12].

Although vertebrate PXR orthologs have been extensively characterized, our understating of this gene in invertebrates, particularly aquatic species, remains limited. The diversity of NR complements across various marine invertebrate phyla is remarkable. These variations might be linked to the wide array of life cycles, developmental strategies, and reproductive adaptations observed among marine invertebrates [17]. The current evolutionary hypothesis regarding NR1I and NR1J homologs suggests that this gene lineage diverged early in the protostome–deuterostome split [18]. An open reading frame encoding a VDR/PXR/CAR-like ortholog (NR1J1β) from the estuarine bivalve peppery furrow shell (*Scrobicularia plana*) was recently isolated and characterized. In this study, NR1J1 was suggested to participate in the detoxification mechanisms of mollusks as its activity was modulated in the presence of the natural toxin okadaic acid (OA) and pesticides in the nanomolar range [18].

Bivalve mollusks constitute an important ecological group of aquatic filter-feeders [19]. Furthermore, filter-feeding behavior is associated with the exposure to and accumulation of a wide variety of natural and human-made chemicals that are potentially harmful to animals, including microalgal biotoxins [20]. Given the anthropogenic pressures they face, it is imperative to safeguard aquatic systems and their communities, especially bivalve populations, and enhance water quality through pollution reduction efforts [21]. It should be considered as a possibility that this unique mode of living and habitat conditions have driven the adaptive evolution of NR1J1 for bivalves to sense and cope with the multiple chemical challenges that come from the environment. This work aims to shed light on the ligand affinity and functions of the four NR1J1 paralogs identified in the marine mussel *Mytilus galloprovincialis*, employing a dual-luciferase reporter assay. Among the substances tested as putative NR1J1 ligand activators were several microalgal toxins and microalgal extracts known to constitute potential chemical challenges in this species.

## 2. Results

**Figure 1:**
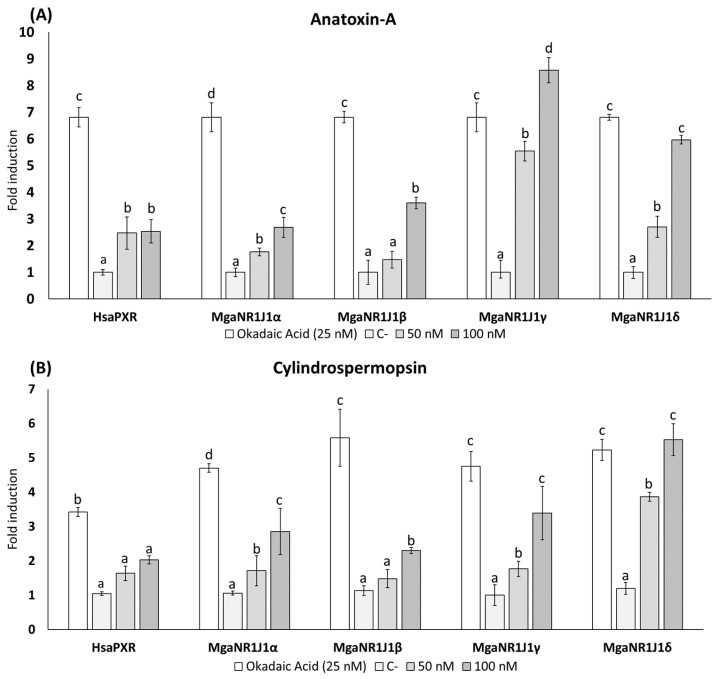
Firefly luciferase transactivation activity mediated by marine mussel NR1J1 paralogs with the freshwater cyanotoxins (**A**) Anatoxin-A and (**B**) Cylindrospermopsin. Human PXR was used as a control assay and OA as a positive control. Values are expressed as mean ± SEM of three replicates. Distinct lowercase letters indicate values significantly different at *p* < 0.01 according to one-way analysis of variance (ANOVA) per gene and Tukey’s multiple range test (*n* = 3).

**Figure 2:**
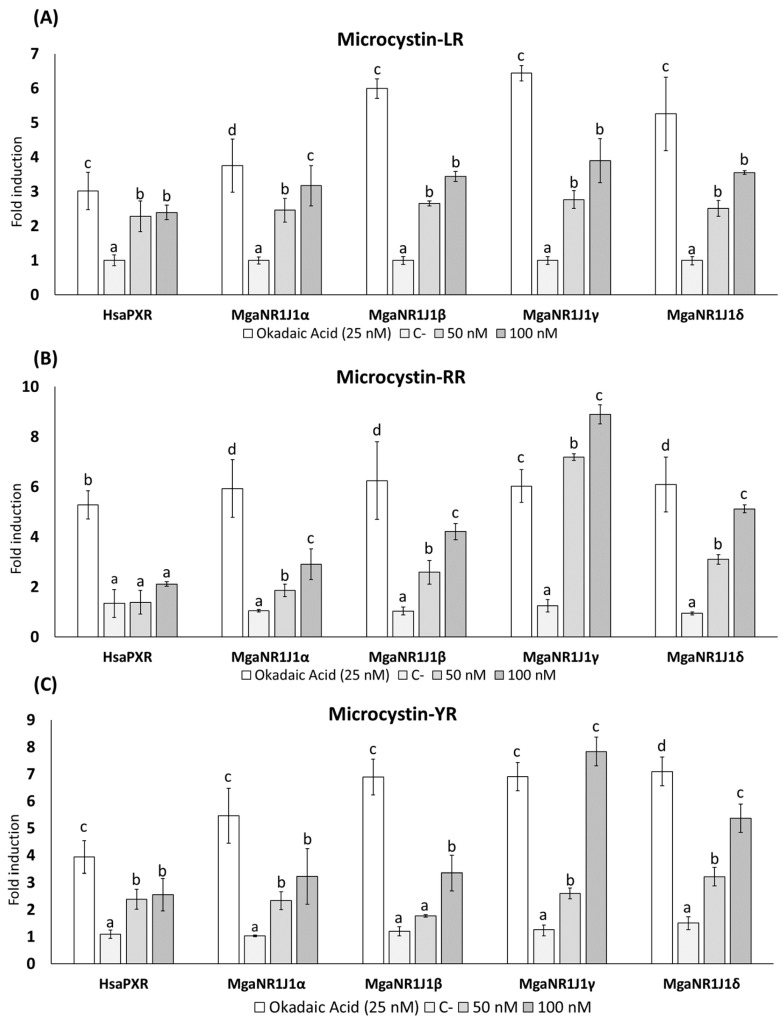
Firefly luciferase transactivation activity mediated by marine mussel NR1J1 paralogs with the freshwater cyanotoxins (**A**) Microcystin-LR, (**B**) Microcystin-RR, and (**C**) Microcystin-YR. Human PXR was used as a control assay and OA as a positive control. Values are expressed as mean ± SEM of three replicates. Distinct lowercase letters indicate values significantly different at *p* < 0.01 according to one-way analysis of variance (ANOVA) per gene and Tukey’s multiple range test (*n* = 3).

**Figure 3:**
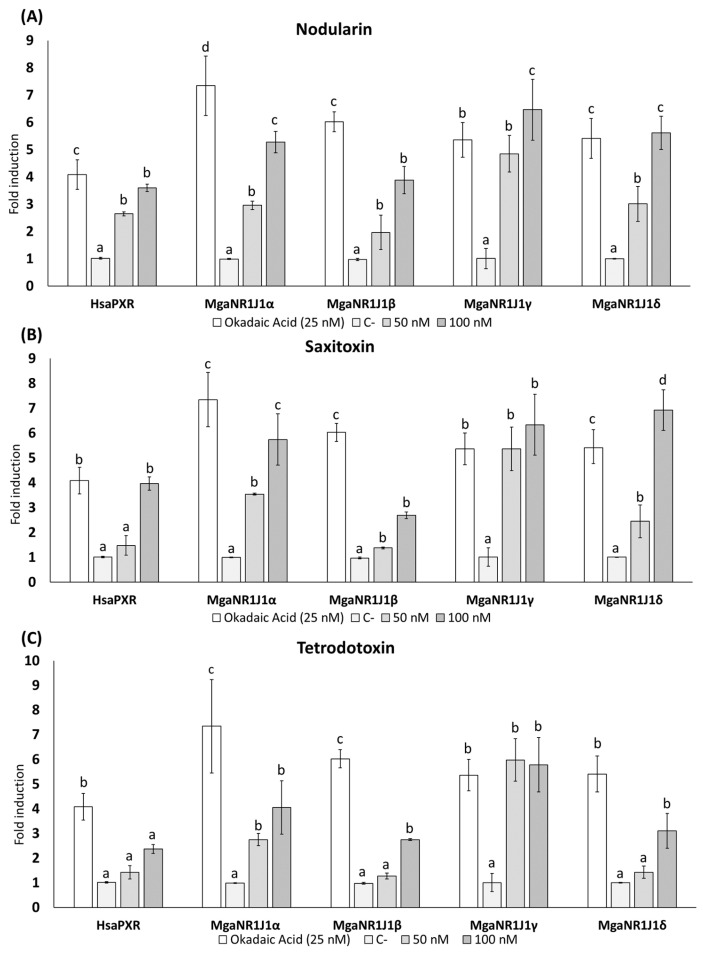
Firefly luciferase transactivation activity mediated by marine mussel NR1J1 paralogs with 3 marine algal toxins: (**A**) Nodularin, (**B**) Saxitoxin, and (**C**) Tetrodotoxin. Human PXR was used as a control assay and OA as a positive control. Values are expressed as mean ± SEM of three replicates. Distinct lowercase letters indicate values significantly different at *p* < 0.01 according to one-way analysis of variance (ANOVA) per gene and Tukey’s multiple range test (*n* = 3).

**Figure 4:**
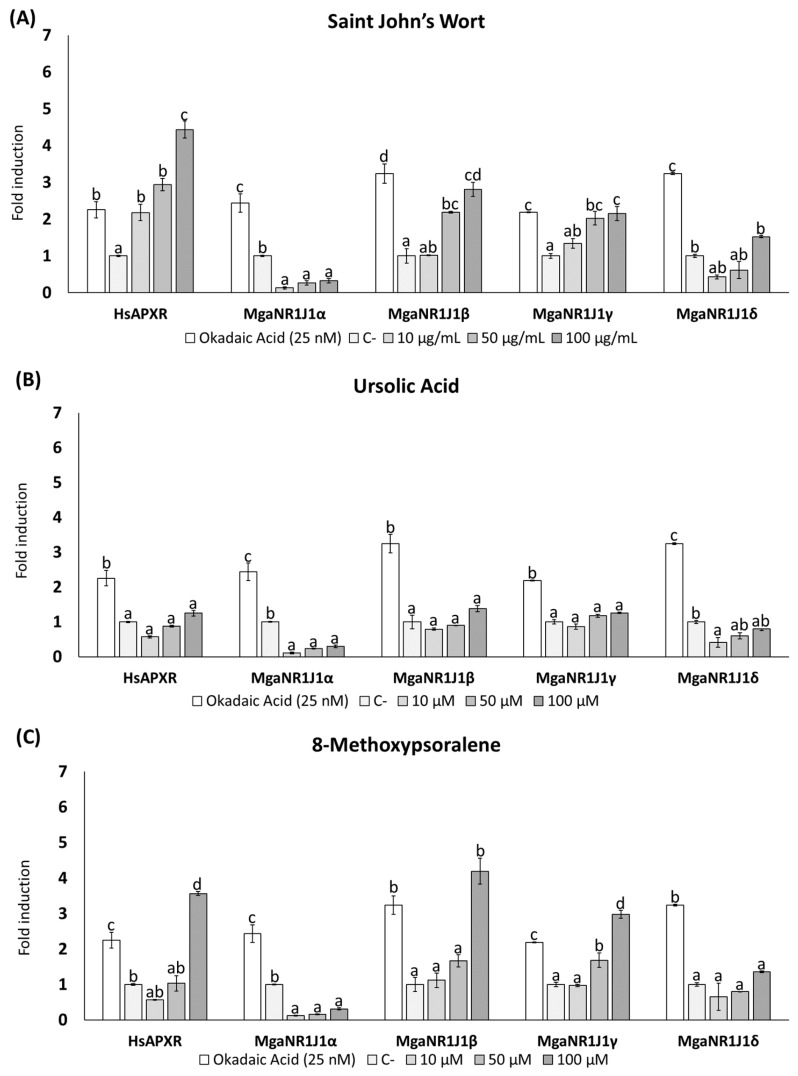
Firefly luciferase transactivation activity mediated by marine mussel NR1J1 paralogs with 3 natural non-toxic compounds: (**A**) Saint John’s Wort, (**B**) Ursolic Acid, and (**C**) 8-Methoxypsoralene. Human PXR was used as a control assay and OA as a positive control. Values are expressed as mean ± SEM of three replicates. Distinct lowercase letters indicate values significantly different at *p* < 0.01 according to one-way analysis of variance (ANOVA) per gene and Tukey’s multiple range test (*n* = 3).

**Figure 5:**
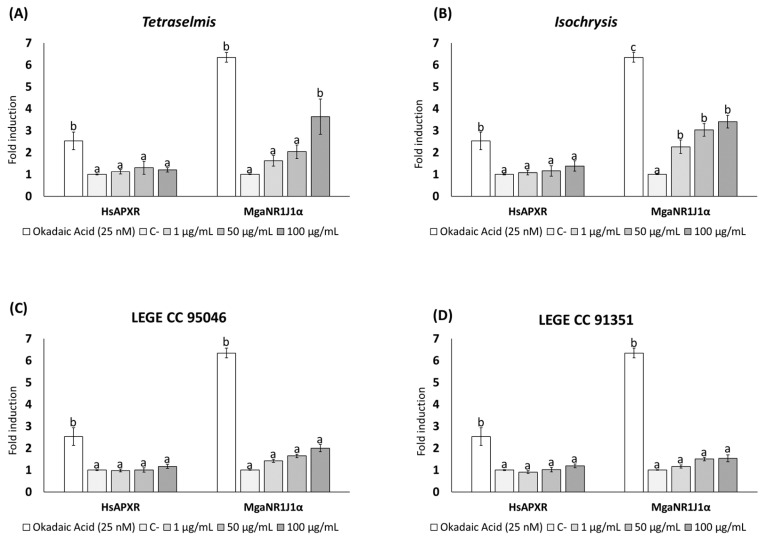
Firefly luciferase transactivation activity mediated by marine mussel NR1J1 α paralog with 4 microalgae extracts: (**A**) *Tetraselmis*, (**B**) *Isochrysis*, (**C**) LEGE CC 95046, and (**D**) LEGE CC 91351. Human PXR was used as a control assay and OA as a positive control. Values are expressed as mean ± SEM of three replicates. Distinct lowercase letters indicate values significantly different at *p* < 0.01 according to one-way analysis of variance (ANOVA) per gene and Tukey’s multiple range test (*n* = 3).

The dual-luciferase reporter gene assay is a method used to evaluate gene expression regulation. The results illustrate the luciferase reporter gene expression levels in terms of the luciferase activity fold change following exposure to freshwater cyanotoxins (Figure 1 and Figure 2), marine toxins (Figure 3), non-toxic natural compounds (Figure 4), and algal extracts (Figure 5).

### 2.1. Freshwater Cyanotoxins

In the presence of both Anatoxin-A (ATX-A) (Figure 1A) and Cylindrospermopsin (CYN) (Figure 1B), an activation of the human PXR (HsaPXR) and marine mussel NR1J1 (MgaNR1J1) receptors was observed. Significant differences were observed in all paralogs at 100 nM ATX-A compared to the negative control. Exposure to 100 nM ATX-A resulted in approximately 9- and 6-fold increases in the luciferase signals of MgaNR1J1γ and MgaNR1J1δ, respectively. In contrast, the induction in the other paralogs studied was lower, reaching up to 4-fold. Notably, no significant differences were observed between the concentrations tested in the human homolog (Figure 1A). Similarly, regarding CYN exposure (Figure 1B), the most sensitive paralogs were MgaNR1J1γ and MgaNR1J1δ. At the highest concentration of CYN tested (100 nM), the signal intensity of these paralogs was comparable to that induced by OA. MgaNR1J1δ exhibited the maximum activity, reaching an approximately 6-fold increase compared to the negative control. In contrast, HsaPXR was the least sensitive paralog. The data obtained from different congeners of Microcystins (MCs) are represented in Figure 2. Overall, MgaNR1J1 paralogs were significantly transactivated in a concentration-dependent manner, while HsaPXR did not respond differently to the concentrations of MCs assayed. Although the results obtained with MC-LR were significantly different, it caused a smaller effect among the congeners studied (Figure 2A). A statistical analysis indicated that the most sensitive paralogs were MgaNR1J1γ and MgaNR1J1δ. Moreover, there were no discernible differences between concentrations except for MgaNR1J1α. Similar trends were obtained when transfected COS-1 cells were exposed to MC-RR (Figure 2B) and MC-YR (Figure 2C). The MgaNR1J1γ paralog was the most sensitive to the exposure to both toxins at 100 nM, exhibiting an increase of up to 9-fold in its activity. Significant differences between the two tested concentrations were observed in all MgaNR1J1 paralogs except for MgaNR1J1α exposed to MC-YR.

### 2.2. Marine Toxins

The exposure to marine toxins resulted in significant transactivation of HsaPXR and MgaNR1J1 paralogs (Figure 3). Notably, the MgaNR1J1γ paralog displayed the highest sensitivity to the effects of these toxins, showing an approximately 6-fold induction at the highest tested concentrations, while the MgaNR1J1β paralog was less responsive. All Nodularin concentrations tested resulted in significant changes compared to the control group (Figure 3A). Moreover, remarkable differences between the concentrations assayed were observed in all groups except for HsaPXR and MgaNR1J1β. Following exposure to 100 nM Saxitoxin (Figure 3B), similarly to MgaNR1J1γ, a significantly high induction (approximately 7-fold) comparable to that with the positive control OA was observed in MgaNR1J1δ, followed by MgaNR1J1α. HsaPXR and MgaNR1J1β showed the lowest activities. Among the concentrations tested, no differences were observed between 50 and 100 nM in MgaNR1J1β and MgaNR1J1γ. Regarding Tetrodotoxin (Figure 3C), high inductions were obtained with both 50 and 100 nM for the paralog MgaNR1J1γ, and significant differences between these concentrations were observed only for MgaNR1J1β and MgaNR1J1δ.

### 2.3. Natural Compounds

The effects on HsaPXR and MgaNR1J1 paralog transactivation by the natural compounds Saint John’s Wort (SJW), Ursolic Acid (UA), and 8-Methoxypsoralene (8M) are shown in Figure 4. The most remarkable effect was the luciferase signal decrease (repression of firefly luciferase expression) with the paralog MgaNR1J1α exposed to these compounds, indicating that they may act as inverse agonists of this paralog.

In the case of SJW (Figure 4A), HsaPXR was the most sensitive (4.43-fold at the highest concentration), with significant concentration-dependent differences compared to the negative control. Significant changes were found for the paralog MgaNR1J1β at the highest concentration, while the MgaNR1J1γ paralog was transactivated significantly at 50 and 100 μg/mL The results obtained with UA are shown in Figure 4B. No significant differences were found among the tested concentrations and the negative control for HsaPXR. Exposure to UA, similarly to that with SJW, led to a significant decrease in luciferase signal for MgaNR1J1α compared to the negative and positive controls. The same was observed with the lowest test concentration for MgaNR1J1δ. No significant differences were found in the transactivation of MgaNR1J1β and MgaNR1J1γ across all the concentrations assayed. Exposure to 8M (Figure 4C) significantly induced a 3.56-fold increase in luciferase activity with HsaPXR at the highest concentration assayed (100 μM). Consistent with the other compounds, a significant decrease in luciferase signal was observed across all concentrations tested for MgaNR1J1α. No significant differences were found in MgaNR1J1δ. However, the MgaNR1J1β and MgaNR1J1γ paralogs had significantly activated luciferase expression with 8M at 100 μM and 50/100 μM, respectively.

### 2.4. Algal Extracts

The transactivation of HsaPXR and MgaNR1J1α upon exposure to different microalgae extracts is shown in Figure 5. No significant changes in luciferase activity induced by HsaPXR were found after exposure to microalgae extracts compared to the negative control. However, *Tetraselmis* and *Isochrysis* extracts significantly increased luciferase activity induced by MgaNR1J1α, while exposure to LEGE CC 95046 and LEGE CC 91351 extracts had no effects. The highest signal was produced by *Tetraselmis* extract (Figure 5A) at the highest concentration (100 μg/mL), with an increase of 3.63-fold induction. Moreover, the transactivation was more evident with the *Isochrysis* extract (Figure 5B), with significant differences at all concentrations tested.

## 3. Discussion

Comprehending the xenobiotic metabolism pathways holds significant importance within the field of toxicology due to their influence on the effects of drugs, xenobiotics, and harmful substances. In mammals and other animals, the nuclear receptor PXR plays a crucial role in regulating the expression of drug-metabolizing enzymes, such as cytochrome P450 enzymes, conjugation enzymes, and transporters [22,23]. As a prototypical nuclear receptor, PXR has a DBD at the N-terminus and an LBD at the C-terminus. This LBD shelters a remarkably plastic ligand-binding pocket, giving PXR the ability to recognize a variety of structurally diverse compounds and a single compound in different orientations [24]. Orthologous genes of PXR (NR1J1) have been reported in marine invertebrates [19,25,26], and recently, four orthologous PXR genes were identified in the marine mussel *Mytilus galloprovincialis* [18,25,26,27]. In the present study, the transfection of four marine mussel NR1J1 paralogs (MgaNR1J1α, MgaNR1J1β, MgaNR1J1γ, MgaNR1J1δ) into the COS-1 cell line coupled to a Gal4 luciferase reporter system was used to facilitate the investigation of NR1J1 functionality and the mechanisms behind the activation and regulation of heterologous metabolism and to draw conclusions regarding their distinct responses and sensitivity to the range of studied toxins and their putative roles in bivalves.

Bivalve feeding on microalgae often leads, in freshwater environments, to the accumulation of cyanotoxins such as MCs and CYN. Molecular-level changes, including modifications in cytoskeleton proteins, disruptions in energy metabolism, and the induction of xenobiotic metabolism enzymes, have been documented in bivalves exposed to cyanotoxins [28,29,30]. However, despite the toxic nature of these compounds, bivalves exhibit a remarkable tolerance towards toxin accumulation and their potential adverse effects, likely due to their ability to rapidly metabolize these toxins. In this sense, Oliveira et al. (2020) [29] concluded that mussels likely possess their own defense mechanisms against cyanotoxins, potentially linked to the overexpression of Enolase 1 (ENO1), Heat Shock Protein 90 (HSP90), and Heterogeneous nuclear ribonucleoprotein A1 (HNRNPA1). 

Based on the obtained data, the marine mussel NR1J1 receptors are sensitive to both freshwater (ATX-A, CYN, MC-LR, MC-RR, and MC-LR) and marine toxins (NOD, SXT, and TTX), especially to the freshwater cyanotoxins ATX-A, MC-RR, and MC-YR (Figure 1, Figure 2 and Figure 3). Evidence has been collected indicating that different types of algal toxins are metabolized and undergo chemical conversion in bivalve tissues. For example, MCs bind covalently with glutathione (GSH), reducing the biological activity of the toxin and facilitating its elimination [31,32,33].

Moreover, this effect was also observed in other aquatic organisms. Li et al. (2013) conducted an experiment in which zebrafish were exposed to crude MCs to examine the role of *mi*RNAs, *cyp*1A1, and PXR in the toxicity of MCs [34]. Their findings suggest that MCs alter the transcription levels of the PXR receptor, implying its role in the metabolism and detoxification of these toxins in zebrafish. Furthermore, in line with our own results, the expression of zebrafish PXR receptor was found to be relatively low in the group exposed to lower concentrations (50 μg/L), while an upregulation was observed in the fish exposed to higher concentrations (200 and 800 μg/L). Given the high toxicity attributed to this specific congener [35], our results further imply that one of its mechanisms of action is linked to these paralogs.

In the marine environment, bivalves face a different array of compounds, such as OA, saxitoxins, domoic acid, and brevetoxins, primarily produced by dinoflagellates. OA and its analogs (DTXs) undergo esterification with fatty acids of varying structures [36,37,38,39] within bivalve tissues, increasing their hydrophilicity and aiding in their elimination [36,37]. Despite the progress in understanding the detoxification of algal toxins, there remains a large gap regarding the molecular processes and enzymes that intervene in and catalyze chemical changes in bivalves. 

The activation of PXR can differ between species, and PXR may exhibit differential activation and regulation of detoxification pathways in response to distinct classes of toxins [26]. Accordingly, in the present work, we studied the differential sensitivity of PXR and its invertebrate orthologs towards various toxins, shedding light on the adaptive responses of bivalves to different environmental challenges and the evolution of detoxification mechanisms in response to specific classes of toxins (Figure 1, Figure 2 and Figure 3).

Our findings demonstrated significant transactivation of both HsaPXR and MgaNR1J1 paralogs upon exposure to marine toxins (Figure 3). Despite the limited information available regarding the interaction of marine toxins with the PXR receptor, certain sterols from the marine sponge *Theonella swinhoei* have been explored for their ability to interact with PXR. Solomonsterol A, a sulfated sterol, represents the first example of a potent human PXR agonist derived from marine sources. It significantly boosted receptor activity by 4–5-fold in transactivation assays at 50 μM with a human hepatocyte cell line (HepG2 cells) and demonstrated increased expression of two well-characterized PXR target genes, *cyp*3A4 and *mdr*1 [40,41].

The transactivation of the PXR receptor involves several mechanisms that can vary depending on the paralog studied [42]. Upon activation by ligands, the PXR receptor undergoes a conformational change, leading to the release of corepressors and the recruitment of co-activators, such as the steroid receptor co-activator, SRC-1 [42]. Theoretically, paralogous genes are expected to exhibit identical expression levels due to their similar sequences and chromatin environments. However, due to selective pressures throughout evolution, functional divergences and expression differences have emerged among paralogous genes [43].

Our study identified MgaNR1J1γ as the paralog most sensitive to the tested toxins, followed by MgaNR1J1δ, with over 7- and 5-fold induction, respectively. However, STX and CYN represent exceptions to this pattern, exerting their most pronounced effect on MgaNR1J1δ, with MgaNR1J1γ following in sensitivity (Figure 1, Figure 2 and Figure 3). The differential sensitivity of various MgaNR1J1 paralogs to different toxins demonstrates a nuanced response mechanism within the organisms. Additionally, this finding suggests that marine mussels may exhibit a consistent activation of NR1J1 paralogs regardless of the origin of the toxins, highlighting the importance of understanding the broader detoxification mechanisms employed by bivalves in response to diverse toxin exposures, irrespective of their habitat.

Moreover, HsaPXR utilized as an assay control can offer insights into the molecular evolution of species. The transactivation of HsaPXR when exposed to the toxins consistently remained lower than the transactivation of MgaNR1J1 paralogs across all cases. This discrepancy suggests potential differences in the evolutionary adaptation of species’ gene functions to suit their respective environments and requirements. The study of orthologous genes provides valuable information in this regard, shedding light on how sequences and functions diverge over time.

Some natural compounds, such as *Hypericum perforatum*, also known as SJW, have been studied as potential agonists of PXR. SJW, a herbal remedy that is widely used in traditional Chinese medicine for treating moderate depression [44,45], exhibited activation of HsaPXR and three of the four MgaNR1J1 paralogs in our study (Figure 4A), consisted with prior research [46,47]. SJW is a sesquiterpenoid containing hyperforin, known for its psychoactive activity [46]. Hyperforin upregulates the gene expression of P450 oxidoreductase and epoxide hydrolase involved in drug metabolism in the human liver and efficaciously activates human PXR [48]. As hyperforin is an abundant, lipophilic component of SJW, it is expected to activate HsaPXR. In that sense, Yan et al. (2021) described the activation of PXR in transfected Caco-2 cells after exposure to different concentrations of hyperforin (0.05–1 μM) [49].

Ursolic Acid (UA) is a pentacyclic triterpenoid derived from the berries, flowers, and fruits of medicinal plants, such as *Rosemarinus officinalis*, that has been extensively studied for its chemopreventive properties, although it has presented other biological effects, such as anti-inflammatory, antiproliferative, antiapoptotic, and antiobesity effects [50,51,52]. Seow and Lau (2017) reported that the major chemical constituents of *Rosmarinus officinalis,* which include UA, carnosic acid, and carnosol, among others, are PXR activators. Particularly, human, mouse, and rat PXR were activated by 30 μM of UA [23]. These results agree with the data obtained in the present work, as we found that UA activated HsaPXR and the marine mussel NR1J1 paralogs MgaNR1J1β and MgaNR1J1γ at similar concentrations (50–100 μM). Nevertheless, Chang et al. (2017) found that in vitro UA (10–20 μM) could act as an inhibitor of HsaPXR. This suggests that further studies are needed to determine the role of UA in activating PXR [53].

Finally, coumarins are other compounds known to bind to PXR. Coumarins are natural compounds known for their toxicity, with the exception of 8-methoxypsoralen, which holds pharmacological value. It is used in combination with UV radiation to treat psoriasis and certain malignant dermatoses [54]. Yang et al. (2007) demonstrated that 8-MOP activated not only HsaPXR but also rat PXR [55]. Following these previous findings, our current study showed that 8-MOP activated HsaPXR and three of the four MgaNR1J1 paralogs.

The results obtained from studying these compounds will contribute not only to the understanding of their mechanism of toxicity but also to proposing innovative biosensors based on metabolic pathways. Given the widespread presence of freshwater and marine toxins, the development of a rapid, sensitive, and reliable method for their detection in environmental samples appears imperative. In this sense, monitoring the detoxification process could be used for the environmental control of these toxins.

## 4. Materials and Methods

### 4.1. Chemical and Reagents

ATX-A, CYN, MC-LR, MC-RR, MC-YR, NOD, SXT, OA, and TTX (≥95% purity) were purchased from Enzo Life Sciences (Lausen, Switzerland) and Cifga Laboratory (Lugo, Spain). The natural compounds (SJW, UA, and 8M) were selected after bibliographic research and purchased from Merck (Darmstadt, Germany).

### 4.2. Microalgae Extraction

The microalgae selected were the marine *Isochrysis* and *Tetraselmis*, both used as feed in aquaculture, and two non-toxic cyanobacteria strains from the LEGE-CC culture collection (https://lege.ciimar.up.pt/) (accessed on 10 October 2022), 91351 (*Microcystis aeruginosa*) and 95046 (*Cylindrospermopsis raciborskii*). To obtain cell extracts, 0.4 g of each microalga (freeze-dried) was weighed and dissolved in 10 mL of milli-Q water. After that, the solutions were ultrasonicated for 15 min and then centrifuged at 4500 rpm for 15 min; the supernatant was collected and stored at −20 °C until its use. *Isochrysis* (PhytoBloom ref. AADISS003) and *Tetraselmis* (PhytoBloom AADTES003) were provided as freeze-dried material from Necton (Olhão, Portugal).

### 4.3. Partial Gene Isolation and Plasmid Vector Construction

Marine mussels, *Mytilus galloprovincialis*, were purchased at the local market (Docapesca), Matosinhos, Portugal. 

Total RNA was extracted from gills with NZYol (NZYTech, Lisbon, Portugal), followed by purification using the RNeasy Mini Kit (QIAGEN, Hilden, Germany)), following the manufacturer’s guidelines. The RNA quantity and integrity were assessed by spectrophotometry (Take3 and Synergy HT Multi-Mode Microplate Reader, Biotek, Agilent, Santa Clara, CA, USA), and electrophoresis (2% agarose gel), and the RNA was stored at −80 °C. The NZY First-Strand cDNA Synthesis Kit (NZYTech, Lisbon, Portugal) was used for cDNA synthesis using 0.5 μg of total RNA.

The hinge region and LBD of the four marine mussel NR1J1 paralogs (MgaNR1J1α, MgaNR1J1β, MgaNR1J1γ, MgaNR1J1δ) were amplified by PCR using specific primers (Table 1) and Phusion Flash High-Fidelity PCR Master Mix (ThermoFisher, Waltham, MA, USA), followed by digestion with BamHI and KpnI, and cloned into pBIND vectors (Promega, Madison, WI, USA, accession number AF264722) to produce fusion proteins with the yeast transcriptional activator GAL4 (GAL4/NRI1J1-LBD), which acts on proximal downstream promoters [56]. The sequences were confirmed by automated Sanger sequencing (Eurofins GATC, Constance, Germany). The sequences were deposited in GenBank (accession numbers OR892305, OR892306, OR892307, and OR892308). The pBIND plasmid containing the construct GAL4/HsaPXR-LBD was kindly provided by Elza Fonseca [57].

### 4.4. Compound Preparation and Testing Concentrations

The microalgae extracts, SJW, UA, and 8M were all resuspended in DMSO. The concentrations for the microalgae extracts were set at 1, 10, and 100 μg/mL. Meanwhile, concentrations of 10, 50, and 100 μg/mL for SJW and 10, 50, and 100 μM for UA and 8M were employed to align with the concentrations tested in prior studies [23,24,52].

Similarly, stock solutions of cyanotoxins were dissolved in MeOH. These compounds underwent testing at final concentrations of 50 and 100 nM to ensure a meaningful comparison with the positive control, OA, at 25 nM.

Each assay included solvent controls, kept below 0.1%, to mitigate any potential confounding effects attributable to the vehicle.

### 4.5. Transfection and Transactivation Assays

COS-1, a fibroblast-like cell line that was isolated from the kidney of an African green monkey (ATCC^®^ CRL-1650), was maintained at 37 °C in an atmosphere containing 5% CO_2_ at 95% relative humidity (CO_2_ incubator). The cells were cultured in Dulbecco’s modified Eagle’s medium (DMEM) (PAN-Biotech, Aidenbach, Bayern, Germany) supplemented with 10% fetal bovine serum (PAN-Biotech, Aidenbach, Bayern, Germany) and 1% penicillin/streptomycin (PAN-Biotech, Aidenbach, Bayern, Germany). The cells were cultured following ATCC recommendations, and experiments were performed with culture passages 5–15.

Before transfection, COS-1 cells were seeded onto 24-well culture plates at a density of 2 × 10^5^ live cells/well. The following day, the cells were transfected with 750 ng of pGL4.35 [luc2P/GAL4UAS/Hygro] luciferase reporter vector (Promega, Madison, WI, USA), which contains five UAS elements upstream of the firefly luciferase reporter gene, and 500 ng of pBIND constructions, using lipofectamine 2000 reagent (Invitrogen, Carlsbad, CA, USA) in Opti-MEM transfection medium (Gibco, Carlsbad, CA, USA), according to the manufacturer’s indications. After 5 h of incubation, the cells were washed with Phosphate Buffer Saline (PAN-Biotech, Aidenbach, Bayern, Germany) and exposed to the test compounds and OA (25 nM) as a positive control in phenol-red-free DMEM supplemented with 10% dextran-coated charcoal-treated serum (Invitrogen, Carlsbad, CA, USA) and 1% penicillin/streptomycin (Invitrogen, Carlsbad, CA, USA) [23,24,52]. The following day, the cells were washed and gently lysed with 100 μL of Passive Lysis Buffer (Promega, Madison, WI, USA) for 15 min at 37 °C and 90 rpm. Firefly and *Renilla* luciferase luminescent activities were assessed using the dual-luciferase assay system according to the manufacturer’s instructions (Promega, Madison, WI, USA) and measured with a Synergy HT Multi-Mode Microplate Reader (Biotek, Agilent, Santa Clara, CA, USA). *Renilla* luciferase, co-expressed with the LBD hybrid proteins, was used as an internal control for transfection efficiency [58]. Each condition was tested in duplicate in three independent assays (*n* = 3).

### 4.6. Data Analysis and Statistics

The transactivation results are expressed as the fold induction, calculated as the ratio between firefly luciferase and *Renilla* luciferase luminescent activities normalized to the negative control (solvent control) ratio. The results are presented as the mean of the normalized values, with bars corresponding to the standard error values. Statistical analysis was performed using the SPSS 27.0 software package (LEAD Technologies, Inc., Chicago, IL, USA), utilizing the firefly and *Renilla* luciferase activities ratio. A one-way analysis of variance (ANOVA) was conducted per gene, followed by Tukey’s multiple range test to identify statistically significant differences among variables. Significant differences were set at *p* < 0.01.

## 5. Conclusions

In conclusion, this study sheds light on the intricate mechanisms of xenobiotic metabolism and detoxification, particularly focusing on the role of marine mussel NR1J1 in responding to a diverse array of compounds. The investigation focused on four NR1J1 paralogs in mussels and how they responded to various freshwater and marine toxins, natural compounds, and microalgal extracts. The findings highlight the exceptional sensitivity of MgaNR1J1γ and MgaNR1J1δ paralogs to specific toxins, suggesting a complex regulatory network within these mussels. Furthermore, this study compared the response of mussel NR1J1 paralogs to that of a similar human gene (PXR). This comparison revealed a striking difference: mussel paralogs displayed considerably higher sensitivity to toxins compared to human PXR. This suggests that mussels have evolved specialized adaptations for detoxification, potentially due to the environmental challenges they face. Overall, this research provides valuable insights into the molecular mechanisms of toxin metabolism in mussels. This knowledge has the potential to improve environmental monitoring, biomarker development for toxin detection, and our understanding of evolutionary adaptations in bivalves. Further investigation into the interactions between NR1J1 paralogs and various toxins will be crucial for advancing our knowledge of how mussels respond to environmental stressors and for developing innovative strategies for managing toxins in aquatic ecosystems.

## Figures and Tables

**Table 1:** List of primers to isolate partial sequences of marine mussel NR1J1 paralogs.

Nuclear Receptor	Oligonucleotide Sequence 5′→ 3′	Tm (°C)
MgaNR1J1α	F: aaaGGATCCaaATGCGTAAAGACTGGATCT	55
	R: aaaGGTACCTTACTTCTGTAAATTGAATACTTC	
MgaNR1J1β	F: aaaGGATCCccATGAGAAAAGAGTACATATTA	
	R: aaaGGTACCTCAAGATTTTTGTGGCAACTC	
MgaNR1J1γ	F: aaaGGATCCacATGAGAAAAGATATGATATTAAAT	
	R: aaaGGTACCTTAACTTGGCAAGTCAAATATCT	
MgaNR1J1δ	F: aaaGGATCCaaATGAGAAAAGAAATGATTCTTG	
	R: aaaGGTACCTCAAGATAGGTTCAAAATTTCCA	

## Data Availability

Data are contained within the article.
